# Neuronal Correlates of Cognitive Control Are Altered in Women With Endometriosis and Chronic Pelvic Pain

**DOI:** 10.3389/fnsys.2020.593581

**Published:** 2020-12-16

**Authors:** Genevieve Z. Steiner, Robert J. Barry, Katherine Wassink, Frances M. De Blasio, Jack S. Fogarty, Adele E. Cave, Sapphire Love, Mike Armour

**Affiliations:** ^1^NICM Health Research Institute and Translational Health Research Institute (THRI), Western Sydney University, Penrith, NSW, Australia; ^2^Brain & Behaviour Research Institute and School of Psychology, University of Wollongong, Wollongong, NSW, Australia

**Keywords:** endometriosis, event-related potentials (ERPs), P3, chronic pain, cognitive control

## Abstract

Endometriosis is a debilitating women's health condition and is the most common cause of chronic pelvic pain. Impaired cognitive control is common in chronic pain conditions, however, it has not yet been investigated in endometriosis. The aim of this study was to explore the neuronal correlates of cognitive control in women with endometriosis. Using a cross-sectional study design with data collected at a single time-point, event-related potentials were elicited during a cued continuous performance test from 20 women with endometriosis (mean age = 28.5 ± 5.2 years) and 20 age- and gender-matched controls (mean age = 28.5 ± 5.2 years). Event-related potential components were extracted and P3 component amplitudes were derived with temporal principal components analysis. Behavioral and ERP outcomes were compared between groups and subjective pain severity was correlated with ERP component amplitudes. No significant behavioral differences were seen in task performance between the groups (all *p* > 0.094). Target P3b (all *p* < 0.034) and SW (all *p* < 0.040), and non-target early P3a (eP3a; all *p* < 0.023) and late P3a (lP3a; all *p* < 0.035) amplitudes were smaller for the endometriosis compared to the healthy control group. Lower non-target eP3a (*p* < 0.001), lP3a (*p* = 0.013), and SW (*p* = 0.019) amplitudes were correlated with higher pain severity scores. Findings suggest that endometriosis-associated chronic pelvic pain is linked to alterations in stimulus-response processing and inhibitory control networks, but not impaired behavioral performance, due to compensatory neuroplastic changes in overlapping cognitive control and pain networks.

## Introduction

Chronic pelvic pain (CPP) lasts longer than 6 months, requires medical intervention, and/or causes functional disability (Howard et al., 2000). The most common cause of CPP in women is endometriosis (24–40%), where cells similar to that of the endometrium grow outside the uterine cavity and create lesions. Endometriosis is associated with a range of pain symptoms such as dysmenorrhea (period pain), dyschezia (pain on bowel motions), dysuria (pain on urination), and dyspareunia (pain during sexual intercourse) (Brown and Farquhar, 2015; Mowers et al., 2016; Whitaker et al., 2016; Johnson et al., 2017). However, there is no clear association between the stage and morphology of endometriosis pathology and the severity and nature of pelvic symptoms, suggesting that other factors may be driving the magnitude and characteristics of CPP (Gruppo Italiano per lo Studio dell'Endometriosi, 2001; Vercellini et al., 2007; Ballard et al., 2010; Zondervan et al., 2018). Endometriosis typically occurs during the reproductive years of women's lives and has profound impacts on physical and mental health, social life, school, work, finances, and sexual relationships (Sepulcri and do Amaral, 2009; Nnoaham et al., 2011; Pluchino et al., 2016; Australian Institute of Health Welfare. *Endometriosis in Australia: Prevalence Hospitalisations*. Canberra: AIHW., 2019), affecting >10% of Australian women with an annual economic burden of >$9 billion AUD (Armour et al., 2019). Surgery to remove lesions often results in pain reoccurrence, suggesting an alternative mechanism underpinning CPP in the absence of endometriosis pathophysiology (Brawn et al., 2014).

Pain transitions from acute to chronic when the increased pain signals in nociceptive pathways lead to sensitization, hyperalgesia, and neuronal hyper-excitability (Boadas-Vaello et al., 2017; Cohen et al., 2017; Meacham et al., 2017; Pace et al., 2018), and is driven by maladaptive plasticity in the central nervous system (CNS) (Boadas-Vaello et al., 2017; Meacham et al., 2017). Women with primary dysmenorrhea demonstrate structural alterations in regional gray matter such as hippocampus, hypothalamus, anterior/posterior cingulate, precuneus, medial prefrontal cortex (Tu et al., 2009). These changes lead to cortical disinhibition, which contributes to the generation of pain and hyperalgesia.

Pain disruptions to the CNS affect cognition (Eccleston and Crombez, 1999). Executive function and control processes associated with goal-directed cognition and behavior (e.g., inhibition, flexibility, adaptability, attentional control) are impaired with chronic pain including low back pain, pancreatic pain, rheumatoid arthritis, musculoskeletal pain, and fibromyalgia (Grace et al., 1999; MacDonald et al., 2000; Dick et al., 2002; Suhr, 2003; Weiner et al., 2006; Moriarty et al., 2011; Hamed et al., 2012; Berryman et al., 2014; Tamburin et al., 2014). The mechanism responsible is thought to be shared/overlapping cortical processing pathways (e.g., anterior cingulate cortex) (Frankenstein et al., 2001; Buffington et al., 2005; Zhao et al., 2006; Walteros et al., 2011), where chronic pain activation competes with cognitive resources due to compensatory neuroplasticity, impacting executive function (Hart et al., 2000; Seminowicz and Davis, 2007; Glass et al., 2011; Moriarty et al., 2011; Simons et al., 2014).

Electroencephalographic event-related potentials (ERPs) probe the neuronal activation underpinning neuroplasticity and executive function. The conglomerate P3 component of the ERP, a large positive deflection elicited ~300–500 ms after stimulus onset, comprising multiple overlapping peaks [P3a, P3b, Novelty P3, Slow Wave (SW)], has been widely explored in the context of acute and chronic pain during cognitive control tasks (Rosenfeld and Kim, 1991; Lorenz and Bromm, 1997; Houlihan et al., 2004) such as the oddball task, which requires participants to identify an infrequent target/deviant stimulus amongst a series of non-target/standard stimuli (Steiner et al., 2013a, 2016a,b).

Cross-sectional studies largely show attenuated P3 amplitudes in people with chronic pain *cf*. controls. For example, reduced auditory oddball and visual multi-source interference task P3 amplitudes are observed in fibromyalgia compared to controls (Ozgocmen et al., 2003; Yoldas et al., 2003; Alanoğlu et al., 2005; Samartin-Veiga et al., 2019). Similarly, auditory oddball P3 amplitudes are reduced in rheumatoid arthritis compared to controls (Hamed et al., 2012; Tomasevic-Todorovic et al., 2015), and inversely correlated with pain intensity (Tomasevic-Todorovic et al., 2015). Some studies report no difference in visual stop-signal task P3 amplitude between people with fibromyalgia and controls (González-Villar et al., 2019), or the opposite relationship (i.e., larger visual oddball P3 amplitudes and positively correlated with pain intensity) following upper limb amputation compared to controls (Karl et al., 2004). P3 latency findings are mixed, with reductions in mixed-pathology chronic pain (visual probe task) (Veldhuijzen et al., 2006), and no differences in fibromyalgia (Ozgocmen et al., 2003; Alanoğlu et al., 2005).

This study was the first that aimed to elucidate the neuronal correlates of cognitive control in women who have endometriosis CPP. Compared to controls, it was hypothesized that women with endometriosis and CPP would have higher pain scores, poorer behavioral performance (Suhr, 2003; Alanoğlu et al., 2005; Moriarty et al., 2011; Berryman et al., 2014; Tamburin et al., 2014) and smaller P3 amplitudes (Ozgocmen et al., 2003; Alanoğlu et al., 2005; Hamed et al., 2012; Tomasevic-Todorovic et al., 2015; Whitaker et al., 2016; Samartin-Veiga et al., 2019), no difference in P3 latencies (Ozgocmen et al., 2003; Alanoğlu et al., 2005), and that P3 amplitudes would inversely correlate with pain severity (Tomasevic-Todorovic et al., 2015); overlapping P3 peaks were explored for the first time with temporal principal components analysis (PCA).

## Materials and Methods

### Participants

Participants were 40 right-handed females: 20 women with endometriosis and CPP, and 20 healthy female controls (HCs) that were individually age matched within 6 months. Participants were recruited from Endometriosis Australia's website and social media platforms, personal and professional networks. Participants in the endometriosis group were reimbursed for their participation with a $50 voucher to cover travel costs, as they were part of a larger clinical trial and were required to attend multiple site visits (baseline data from the first site visit where no intervention was given is reported here) (Armour et al., 2018); no reimbursement was provided for HCs. Written informed consent was obtained prior to testing, and the research was approved by the Western Sydney University Human Research Ethics Committee and the Combined Illawarra Area Health/University of Wollongong Human Research Ethics Committee. The protocol encompassing this study was registered with the Australian New Zealand Clinical Trials Registry (ACTRN12617000053325).

### Eligibility Criteria

All participants had a regular menstrual cycle (21–35 days), no history of severe concussions or head trauma, were not currently pregnant, did not experience epilepsy, and had no major neurological or psychiatric conditions. Participants abstained from caffeine and alcohol within 12 h of testing, and tobacco within 6 h of testing. All participants completed a 4-week pain diary to gauge average daily pain ratings and to screen for endometriosis in the HCs as this condition is often undiagnosed (Nnoaham et al., 2011); HCs with a daily pain score >6/10 were excluded. All women with endometriosis had received a diagnosis via laparoscopy within the past 5 years and self-reported their endometriosis stage in accordance with the revised American Fertility Society classification of endometriosis (Rock, 1995): 1 = minimal; 2 = mild; 3 = moderate; 4 = severe. They also subjectively reported CPP and at least one of either dysmenorrhea, dyspareunia, dyschezia, or dysuria throughout the pain diary. The HC group had no current acute injuries or other painful conditions and no history of chronic pain.

### Procedure

After completing the pain diary prior to testing, all participants presented for testing on days 4–11 of their menstrual cycle (within 1 week of completing the pain diary) to control for hormonal changes which may influence ERPs or level of CPP in the endometriosis group; if the participant was on hormonal contraception they were able to complete the test on any day (Johnston and Wang, 1991; Kluck et al., 1992; Tasman et al., 1999; O'Reilly et al., 2004). Testing for this study took place on a single site visit at the HEADBOX and NICM Neurocognition Lab at Western Sydney University, Campbelltown campus. After providing written informed consent, participants completed a standard electroencephalography (EEG) screening questionnaire, the Edinburgh Handedness Inventory, and were fitted with EEG recording equipment and seated approximately 80 cm from a 21-inch screen (LG Flatron W2253TQ) with a keyboard placed in front of them before completing a brief electro-oculogram (EOG) calibration task (Croft and Barry, 2000) and a continuous performance test (AX-CPT).

### AX-CPT Task

The AX variant of the CPT (Rosvold et al., 1956) is a cognitively demanding visual-attention task which uses a contextual cue to investigate attention, processing speed, and executive functions including inhibition, interference control, and response activation (Servan-Schreiber et al., 1996), and reliably elicits the conglomerate P3 complex (Dias et al., 2003; Berryman et al., 2014). This task requires an individual to respond to the *target* letter X but only if it is preceded by the *cue* letter A. Stimuli consisting of the letters A, X, B, and Y were presented in a quasi-random sequence. Participants were instructed to respond via a number pad key press (1) with their dominant hand, as quickly and accurately as possible to the target letter X when it was preceded by the cue letter A. All stimuli were presented for a duration of 250 ms at a fixed inter-stimulus interval (ISI) of 1,200 ms. The probability of correct AX pairings (cued targets) was 70% to develop a pre-potent motor response. Incorrect AY (cued non-target), BX (uncued target) and BY (uncued non-target) pairings were each presented at a probability of 10%. A total of 500 stimuli were presented (AX = 350, AY = 50, BX = 50, and BY = 50); only cued-target AX and cued-non-target AY stimulus pairs were assessed.

Display and stimulus markers were controlled by a separate Dell Optiplex 760 computer using Compumedics Stim2 (4.0.09302005) software. The task was carried out in 2 blocks (approximately 5 min each), with a self-timed break in between, and a brief practice beforehand. Participants with endometriosis also completed other tasks as part of a larger study (Armour et al., 2018); these are not reported here.

### EEG Recording

Continuous EEG was recorded from a 62-channel electrode cap and M2 (Fp1, Fpz, Fp2, AF3, AF4, F7, F5, F3, F1, Fz, F2, F4, F6, F8, FT7, FC5, FC3, FC1, FCz, FC2, FC4, FC6, FT8, T7, C5, C3, C1, Cz, C2, C4, C6, T8, TP7, CP5, CP3, CP1, CPz, CP2, CP4, CP6, TP8, P7, P5, P3, P1, Pz, P2, P4, P6, P8, PO7, PO5, PO3, POz, PO4, PO6, PO8, O1, Oz, O2, CB1, CB2). Cap electrodes were referenced online to M1, and grounded by an electrode positioned between Fpz and Fz. EOG was recorded from electrodes placed 2 cm above and below the left eye for vertical movements, and electrodes placed on the outer canthus of each eye for horizontal movements. All electrodes were sintered Ag/AgCl, and impedances were kept below 10 KΩ. Data were acquired from DC−70 Hz, with a 50 Hz notch filter, and were digitized at 1,000 Hz using Compumedics Neuroscan Synamps2 digital signal-processing system and Neuroscan 4.5.1 Acquire software.

### Data Pre- and Post-processing, Quantification, and Extraction

EEG data were corrected offline for eye movements using the Revised Aligned-Artifact Average (RAAA) procedure (Croft and Barry, 2000), digitally re-referenced to linked mastoids, and low-pass filtered (30 Hz, FIR zero phase shift, 24 dB/Octave). CB1 and CB2 were omitted from all analyses due to excessive noise, leaving 60 electrodes in the montage. For each trial, data were epoched −100 ms to 900 ms relative to stimulus onset, and baseline corrected to the pre-stimulus period (−100 to 0 ms). Trials with extreme amplitudes (outside ±100 μV), omission or commission errors were rejected. AX trials were also rejected if they did not have valid reaction times within ± 2 *SD* of the intra-individual mean. The remaining epochs were used to generate ERPs to target (AX) and non-target (AY) trials.

ERP data were down-sampled to 250 Hz and submitted to four separate unrestricted temporal PCAs: one for each group (endometriosis, HC) and condition (target, non-target). Separate PCAs were used as this is recommended to reduce misallocation of ERP variance when ERP latencies, amplitudes and/or topographies are expected to vary between groups and/or conditions (Barry et al., 2016). Input for each PCA was 1,200 cases (20 participants × 60 scalp electrode sites) and 250 variables (time points), producing a case:variable ratio of 4.8. All PCAs were conducted in MATLAB (the Mathworks, v 8.0, R2012b) with Dien's (2010) ERP PCA toolkit (v. 2.23) using the covariance matrix with Kaiser normalization, and all 250 factors underwent unrestricted Varimax rotation to optimally distribute error variance (Kayser and Tenke, 2003).

PCA factors explaining more than 1.5% of the ERP variance were reconstituted into “virtual” ERPs and correlated at the midline sites (Cz, Fz, and Pz) with the grand mean raw ERP waveforms to ensure a good fit. P3 components were identified based on factor loadings, latency, topography, polarity, and sequence. For each condition, the temporal correspondence between P3 components in each group was assessed using the congruence coefficient (*r*_*c*_) (Tucker, 1951), which is a non-standardized correlation of the unscaled factor loadings; *r*_*c*_ ≥.95 indicates component equivalence, and 0.95 > *r*_*c*_ ≥ 0.85 indicates component similarity (Lorenzo-Seva and ten Berge, 2006). Topographical similarities were assessed using Pearson's correlations across the scalp electrodes.

### Statistical Analysis

Demographics, pain severity (calculated as the average pain score out of 10 for each participant across 28 days of pain diary entries), and AX-CPT performance (reaction time, omission, and commission errors) were compared with between-groups *t*-tests. Separate mixed-model MANOVAs compared P3 component amplitudes for the between-subjects factor Group (endometriosis vs. HC), and within-subjects factor Condition (target vs. non-target), and the Sagittal [frontal (F3, Fz, F4), central (C3, Cz, C4), parietal (P3, Pz, P4)] and Coronal [left (P3, C3, P3), midline (Fz, Cz, Pz), right (F4, C4, P4)] topographic planes. Planned orthogonal contrasts in the sagittal plane compared frontal vs. parietal, and mean frontal/parietal vs. central regions; and in the coronal plane they compared the left vs. right hemispheres, and left/right hemispheric mean vs. midline region. Correlations were conducted between maximal P3 component amplitudes (selected from the 9 analyzed sites) and pain severity across the groups, and between component amplitude and pain duration (defined as time since first chronic pain symptom), and endometriosis staging and pain severity within the endometriosis group.

To detect a large effect size (Cohen's *d* = 0.80, eta^2^ = 0.13) at 80% power, α = 0.05, one-tailed, 20 participants per group were required. All *t*-tests had (38) *df* and *F*-tests had (1, 38) *df*, and all were tested against one-tailed α = 0.05. Greenhouse-Geisser correction was not applied as tests utilized single degree of freedom contrasts, and univariate MANOVA does not require sphericity (O'Brien and Kaiser, 1985; Vasey and Thayer, 1987). No Bonferroni-type alpha adjustments were made for the MANOVAs because contrasts were planned, and the number of contrasts was less than the degrees of freedom (Tabachnick and Fidell, 1989).

## Results

### Participant Characteristics

Participant characteristics including age, sex, pain severity and duration, and self-reported endometriosis stage, comorbidities, and medication use are detailed in Table 1; five participants could not recall diagnosed endometriosis stage.

**Table 1:** Participant characteristics.

	**Endometriosis**	**HCs**	***t*-test**
*N*	20	20	
Mean years of age ± SD (range)	28.5 ± 5.2 (21–41)	28.5 ± 5.2 (21–41)	*p* = 0.956
Mean pain severity ± SD (range)	4.1 ± 1.5 (1.1–7.2)	0.2 ± 0.2 (0.0–0.7)	*p* < 0.001
Mean years pain duration ± SD (range)	11.8 ± 5.4 (2–21)	N/A	
Mean endometriosis stage ± SD (range)	3.1 ± 1.0 (2–4)	N/A	
Comorbidities (*N* participants)			
Chronic fatigue syndrome	6	0	
Migraine	8	0	
Depression	5	0	
Anxiety	1	2	
ADHD	1	0	
Asperger's syndrome	1	0	
Medication Use (*N* participants)			
Oral contraceptive pill	3	6	
Hormonal IUD	6	0	
GnRH agonists	1	0	
OTC analgesics^*^	19	9	
Benzodiazepines	1	0	
Opioids	7	0	
Gabapentinoids	3	0	
Antidepressants	3	1	
Stimulants	1	0	
Levothyroxine	0	1	
Antifibrinolytics	1	0	
Proton Pump Inhibitors	1	0	
Biguanides	1	0	

* *Over the counter (OTC) analgesics included regular use of paracetamol/acetaminophen and non-steroidal anti-inflammatories (NSAIDs) as reported in daily pain diaries and separately to manage pelvic pain*.

### Behavioral Outcomes

Table 2 shows the percentage of accepted target and non-target trials, omission and commission errors, mean reaction times and their standard deviations; these did not differ between groups.

**Table 2:** Mean behavioral results ± SD and statistical difference.

	**Endometriosis**	**HCs**	***t*-test**
Accepted target trials %	91.6 ± 4.1	91.4 ± 7.0	*p* = 0.406
Accepted non-target trials %	81.2 ± 16.8	82.0 ± 12.1	*p* = 0.296
Omission errors %	1.5 ± 2.1	0.9 ± 1.4	*p* = 0.125
Commission errors %	18.8 ± 16.8	13.8 ± 9.5	*p* = 0.127
Reaction time ms	348.9 ± 64.1	325.8 ± 51.9	*p* = 0.094

### PCA Outcomes

**Figure 1:**
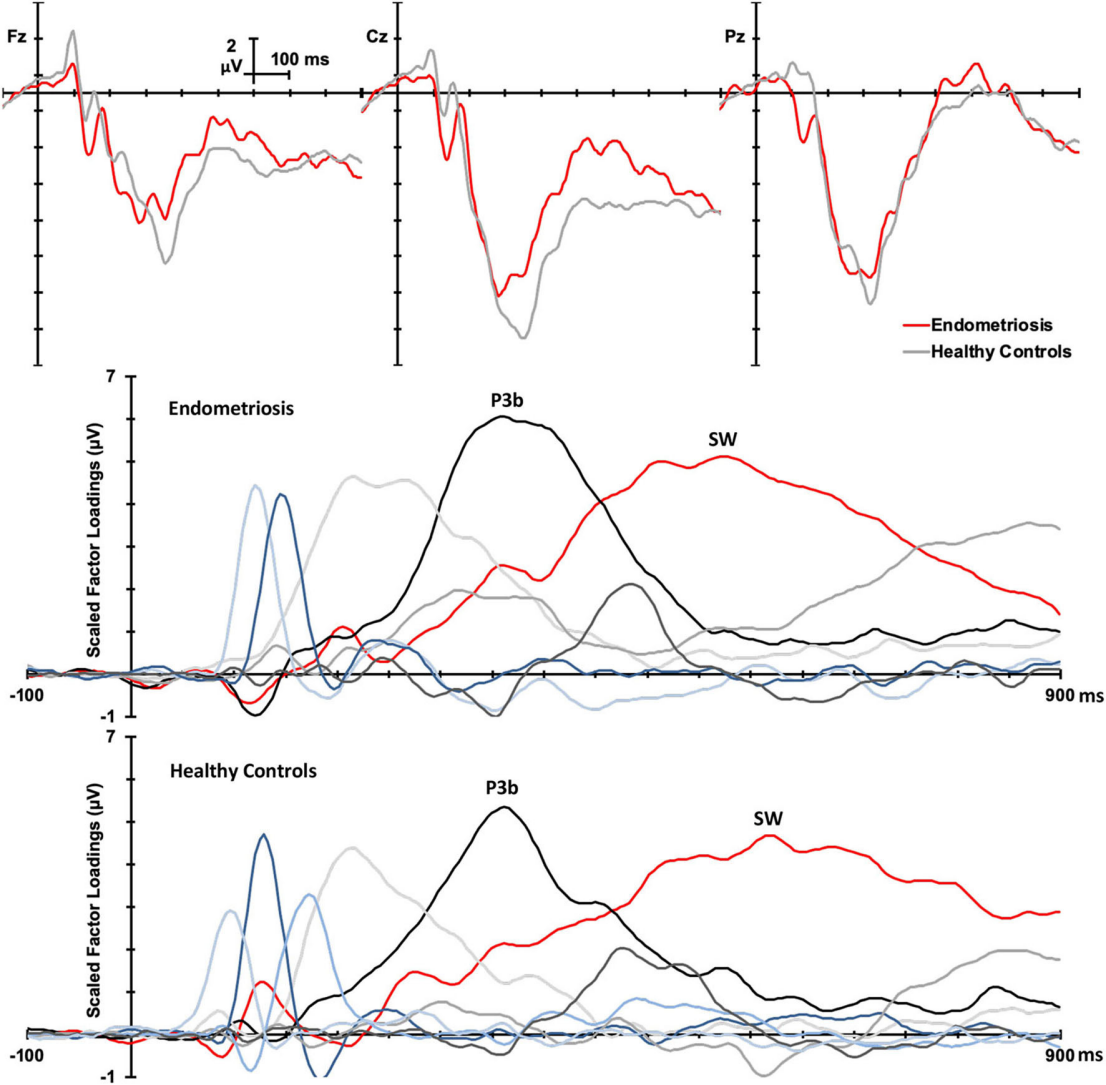
**(Top)** Grand mean ERPs (with negative plotted upwards) to target stimuli for the endometriosis (red) and HC (gray) groups across three midline sites (Fz, Cz, Pz). **(Middle)** Factor loadings for the 7 PCA components for the endometriosis group, the identified P3b (TF02) and SW (TF01) components are marked. **(Bottom)** Factor loadings for the 8 PCA components for the HC group; again P3b (TF02) and SW (TF01) are labeled.

Grand mean ERPs to target stimuli at midline sites for the endometriosis and HC groups are shown in the top panel of Figure 1. For all target components meeting the 1.5% variance cut-off, Table 3 lists their latency and variance explained (in variance order) for the endometriosis and HC groups; the corresponding factor loading plots are shown in the middle and lower panels of Figure 1. A good fit was found between the raw and reconstituted data at the midline sites across groups, *r*_(248)_ ≥ 0.97, *p* < 0.001. The first two factors of each target PCA were identified as P3b (TF02) and SW (TF01). The other factors were outside the scope of this investigation and will not be discussed further. The component time courses were equivalent for P3b (*r*_*c*_ = 0.97) and SW (*r*_*c*_ = 0.96) between the endometriosis and HC groups, as were their topographies: P3b *r*(58) = 0.86, *p* < 0.001; SW *r*(58) = 0.88, *p* < 0.001.

**Table 3:** Target PCA outcomes for the endometriosis and HC groups.

**Endometriosis group**									
P3 Component	SW	P3b							
Factor	TF01	TF02	TF03	TF04	TF05	TF06	TF07		Total factors = 7
σ^2^ %	33.9	25.8	14.6	11.8	3.1	2.6	1.5		Total σ^2^ = 93.3
Latency ms	572	356	212	864	120	144	484		
**HC Group**									
P3 Component	SW	P3b							
Factor	TF01	TF02	TF03	TF04	TF05	TF06	TF07	TF08	Total factors = 8
σ^2^ %	42.9	23.6	12.3	4.1	3.2	3.1	2.3	1.8	Total σ^2^ = 93.3
Latency ms	616	360	212	128	864	172	474	96	

**Figure 2:**
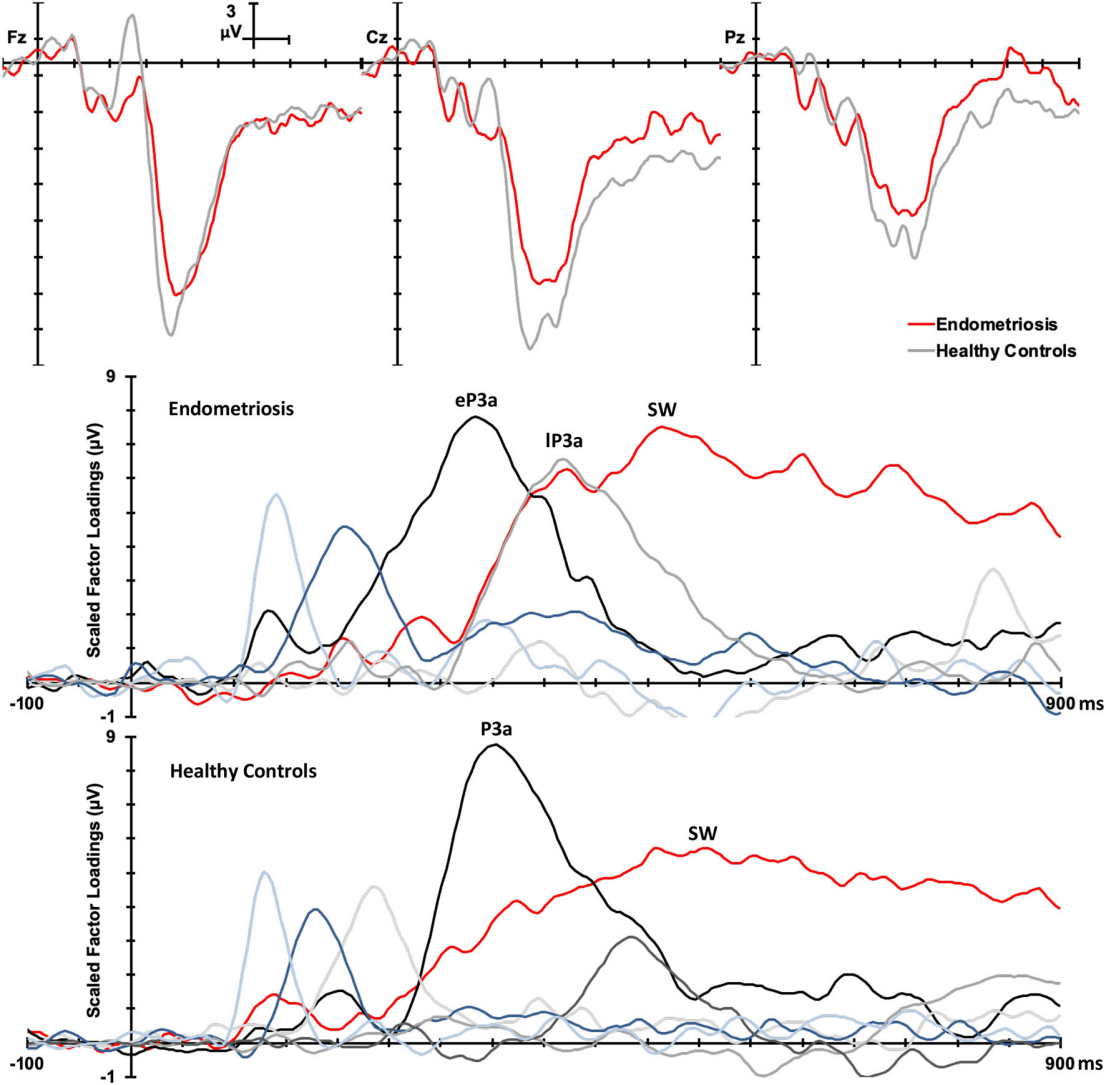
**(Top)** Grand mean ERPs (with negative plotted upwards) to non-target stimuli for the endometriosis (red) and HC (gray) groups at three midline sites (Fz, Cz, Pz). **(Middle)** Factor loadings for the 6 PCA components for the endometriosis group, with the identified eP3a (TF02), lP3a (TF03), and SW (TF01) components labeled. **(Bottom)** Factor loadings for the 7 PCA components for the HC group, with the P3a (TF02) and SW (TF01) components marked.

Non-target grand mean ERPs are shown in the top panel of Figure 2, and the PCA factor loadings for the endometriosis and HC groups are shown in the middle and lower panels, respectively. Latency and variance explained are shown in Table 4 for each PCA to non-target stimuli; across groups, the raw and reconstituted PCA non-target data were a good fit at midline sites, *r*(248) ≥ 0.98, *p* < 0.001. The first three factors of the non-target PCA for the endometriosis group were identifiable as early P3a (eP3a; TF02), late P3a (lP3a; TF03), and SW (TF01) components, and the first two factors for the HC group as P3a (TF02) and SW (TF01); see Figure 2. Again, all other factors were excluded as they were not part of the P3 late positive complex. When the single P3a component for HCs, was compared with the eP3a and lP3a in the endometriosis group, their time courses approached similarity (eP3a: *r*_*c*_ = 0.82; lP3a: *r*_*c*_ = 0.83), and their topographies corresponded well: eP3a *r*(58) = 0.63, *p* < 0.001; lP3a *r*(58) = 0.95, *p* < 0.001. Temporal (*r*_*c*_ = 0.99) and topographic (*r*(58) = 0.79, *p* < 0.001) equivalence was found between groups for the identified SW components.

**Table 4:** Non-target PCA outcomes for the endometriosis and HC groups.

**Endometriosis group**								
P3 Component	SW	eP3a	lP3a					
Factor	TF01	TF02	TF03	TF04	TF05	TF06		Total factors = 6
σ^2^ %	46.4	18.3	12.3	5.0	3.5	1.8		Total σ^2^ = 87.3
Latency ms	512	332	416	208	140	832		
**HC Group**								
P3 Component	SW	P3a						
Factor	TF01	TF02	TF03	TF04	TF05	TF06	TF07	Total factors = 7
σ^2^ %	42.5	29.5	4.5	3.0	2.8	2.8	2.5	Total σ^2^ = 87.6
Latency ms	508	352	236	128	180	292	484	

### Group Differences in ERP Components

**Figure 3:**
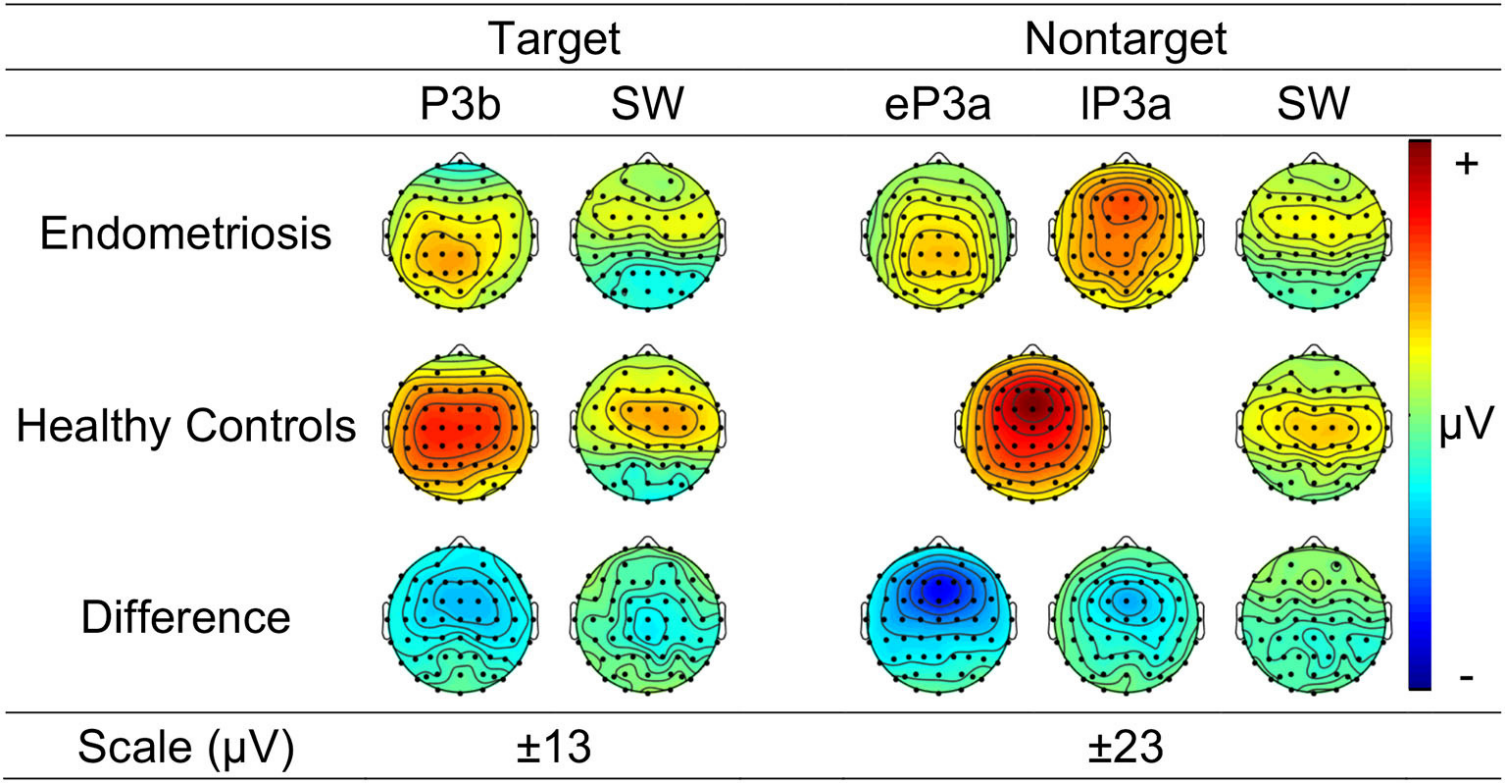
Topographic headmaps for the endometriosis **(Top)** and HC **(Middle)** groups, and their difference (**Bottom**; endometriosis minus HC) for each of the assessed P3 components.

Figure 3 shows the grand mean headmaps in each group for target P3b and SW, non-target P3a (HC only), eP3a and lP3a (endometriosis only), and SW; between group differences (endometriosis relative to HC) are shown in the bottom row. Table 5 displays the corresponding statistics with the direction of effects denoted with the qualifiers “<” and “>” signaling less than and greater than, respectively, and interactions between the contrasts as “×.” Target P3b had virtually identical latencies for endometriosis (356 ms) and HC (360 ms) groups; this is one datapoint difference as data were downsampled to 250 Hz. Across groups P3b was larger centrally than fronto-parietally, in the left than right hemisphere, and parietally in the left hemisphere and midline. As shown in Figure 3 and Table 5, P3b was smaller in the endometriosis than HC group, particularly centrally. Target SW peaked earlier in the endometriosis (572 ms) than HC group (616 ms). Target SW had a fronto-central and central-right topography across the groups. SW's central-right topography was smaller for the endometriosis group compared to HCs.

**Table 5:** Statistical outcomes for target and non-target P3 component amplitudes.

**Component**	**Effect**	***F***	***p***	**$\eta_{\text{p}}^{2}$**
**TARGETS**				
**P3b**	Central > Frontal/Parietal	38.42	<0.001	0.50
	Left > Right	8.45	0.006	0.18
	Frontal < Parietal × Left > Right	10.57	0.002	0.22
	Frontal < Parietal × Midline > Left/Right	7.31	0.010	0.16
	Endometriosis < Healthy Controls	4.83	0.034	0.11
	Endometriosis < Healthy Controls × Central > Frontal/Parietal	5.52	0.024	0.13
**SW**	Frontal > Parietal	21.02	<0.001	0.36
	Central > Frontal/Parietal	110.67	<0.001	0.74
	Central > Frontal/Parietal × Left < Right	46.59	<0.001	0.55
	Endometriosis < Healthy Controls × Central > Frontal/Parietal	6.23	0.017	0.14
	Endometriosis < Healthy Controls × Central > Frontal/Parietal × Left < Right	4.52	0.040	0.11
**NON-TARGETS**				
**eP3a**	Central > Frontal/Parietal	13.37	<0.001	0.26
	Midline > Left/Right	33.58	<0.001	0.47
	Frontal > Parietal × Midline > Left/Right	10.34	0.003	0.21
	Central > Frontal/Parietal × Midline > Left/Right	12.36	0.001	0.25
	Endometriosis < Healthy Controls	15.79	<0.001	0.29
	Endometriosis < Healthy Controls × Frontal > Parietal	12.11	0.001	0.24
	Endometriosis < Healthy Controls × Midline > Left/Right	12.71	0.001	0.25
	Endometriosis < Healthy Controls × Frontal > Parietal × Midline > Left/Right	11.19	0.002	0.23
	Endometriosis < Healthy Controls × Central > Frontal/Parietal × Midline > Left/Right	5.57	0.023	0.13
**lP3a**	Frontal > Parietal	14.59	<0.001	0.28
	Central > Frontal/Parietal	6.73	0.013	0.15
	Midline > Left/Right	47.53	<0.001	0.56
	Frontal > Parietal × Left < Right	7.75	0.008	0.17
	Frontal > Parietal × Midline > Left/Right	8.42	0.006	0.18
	Endometriosis < Healthy Controls	5.80	0.021	0.13
	Endometriosis < Healthy Controls × Central > Frontal/Parietal	8.48	0.006	0.18
	Endometriosis < Healthy Controls × Frontal > Parietal × Midline > Left/Right	4.80	0.035	0.11
	Endometriosis < Healthy Controls × Central > Frontal/Parietal × Midline > Left/Right	5.81	0.021	0.13
**SW**	Frontal > Parietal	6.80	0.013	0.15
	Central > Frontal/Parietal	111.99	<0.001	0.75
	Central > Frontal/Parietal × Left < Right	5.55	0.024	0.13

*Direction of effects is denoted with the qualifiers “<” and “>,” and interactions between contrasts as “×.” Frontal/Parietal = fronto-parietal mean; Left/Right = hemispheric mean*.

Non-target P3a for HCs peaked at 352 ms and the equivalent components for the endometriosis group peaked before and after at 332 ms (eP3a) and 415 ms (lP3a). As shown in Table 5, across groups the P3a (HC) and eP3a (endometriosis) components had a central, midline, and fronto-central midline distribution. Non-target eP3a (endometriosis) was smaller than P3a (HC) globally, and regionally in the fronto-midline, and fronto-parietally in the midline; see Figure 3 and Table 5. The P3a (HC) and lP3a (endometriosis) had a fronto-central and midline distribution, with relatively larger amplitudes in the frontal-midline and frontal-right regions. The lP3a (endometriosis) was globally smaller than P3a (HC), particularly in the frontal-midline and at the vertex. Non-target SW had virtually identical peak latencies across the groups (endometriosis = 512 ms; HC = 508 ms), differing by one data-point only. This component demonstrated a fronto-central and central-right topography; there was no statistically significant difference between groups.

### P3 Components and Pain

The maximal site from the nine electrodes analyzed was selected for each component to enter into correlations. For the targets, these were Pz and Cz, respectively, for the endometriosis and HC P3b components; and Fz for the target SW in both groups. For the non-targets, Cz and Fz were selected for the endometriosis eP3a and lP3a components, respectively, and Fz was selected for the HC P3a; Fz was selected for the endometriosis group, and Cz for the HC group when assessing the non-target SW.

**Figure 4:**
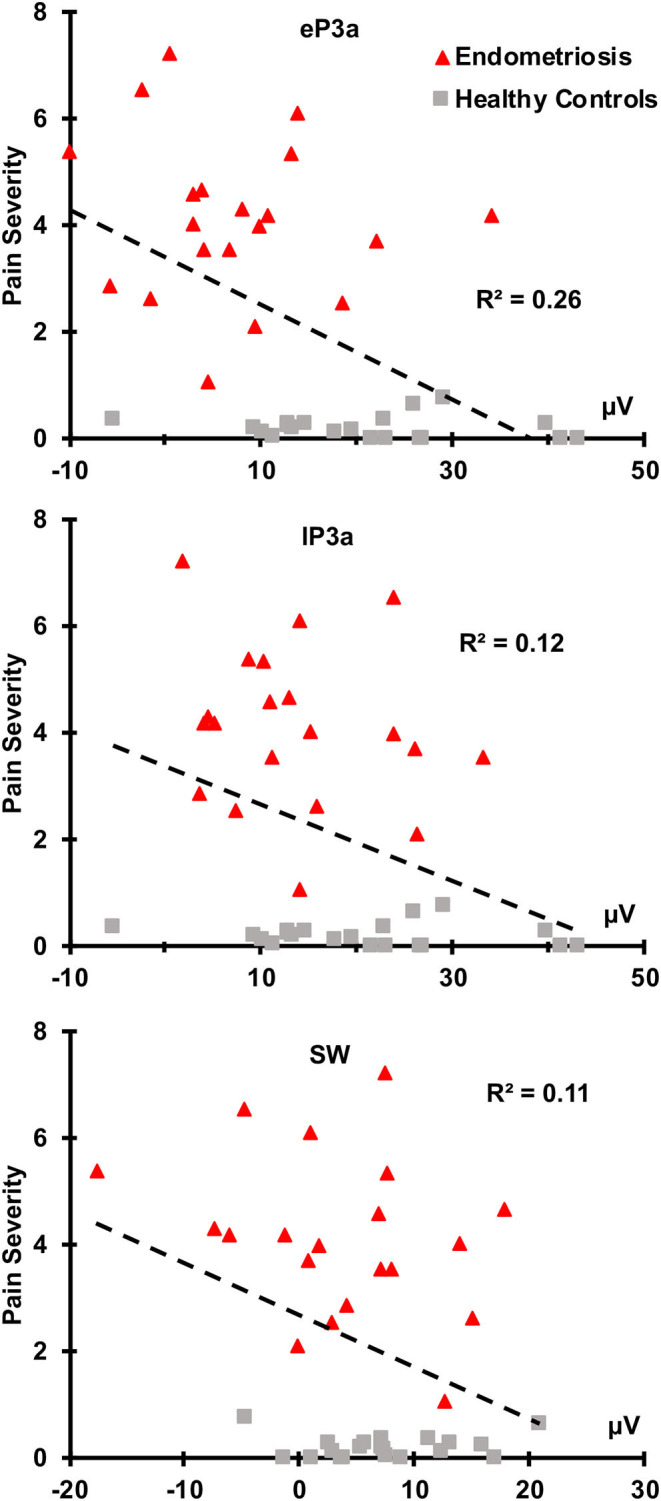
Nontarget eP3a, lP3a, and SW were inversely correlated with pain severity across the endometriosis (red) and HC (gray) groups.

There were no relationships between the target P3b or SW and pain severity (all *p* ≥ 0.050). As shown in Figure 4, greater non-target eP3a (*r*(38) = −0.51, *p* < 0.001), lP3a (*r*(38) = −0.35, *p* = 0.013), and SW (*r*(38) = −0.33, *p* = 0.019) amplitudes were each associated with lower pain severity across the groups. According to Cohen (1988, 1992), *r*-values around 0.1 are considered small, 0.3 are medium, and 0.5 are large, thus pain severity and eP3a had a large effect size, and pain severity and both lP3a and SW had medium effect sizes. There were no significant correlations between ERP component amplitudes and pain duration in the endometriosis group; nor was there a relationship between endometriosis stage and pain severity (all *p* ≥ 0.100).

## Discussion

This study investigated the neuronal correlates of cognitive control in women with endometriosis and CPP, compared to HCs, using P3 ERP components elicited in an AX-CPT. Using PCA, we delineated two P3 components to target stimuli for both groups (P3b and SW), three P3 components to non-targets for the endometriosis group (eP3a, lP3a, and SW), and two non-target components for the HC group (P3a and SW). As hypothesized, target P3b and SW amplitudes were smaller in the endometriosis than HC group, and non-target eP3a and lP3a component amplitudes in the endometriosis group were smaller than that in the single P3a component in the HC group; non-target SW and task performance did not differ significantly between the groups. Component latencies were nearly identical across the groups for target P3b and non-target SW as predicted, however, the target SW was earlier for the endometriosis than HC group, and the non-target eP3a and lP3a peaks in the endometriosis group fell either side of HC group's P3a. As expected, non-target eP3a, lP3a, and SW amplitudes were each inversely associated with lower pain severity scores across the groups; target P3b and SW were not related to pain severity, and no P3 component was associated with pain duration for the endometriosis group.

The behavioral performance (RTs, commission, and omission errors) on the AX-CPT did not differ between women with endometriosis and HCs. These results contradict the majority of previous studies reporting reduced cognitive control in individuals with chronic pain from a variety of conditions (MacDonald et al., 2000; Suhr, 2003; Moriarty et al., 2011; Berryman et al., 2014). Nevertheless, Glass et al. (2011) documented no behavioral differences between people with fibromyalgia and HCs whilst observing clear functional brain differences via fMRI. Findings may be due to compensatory neuroplasticity, preserving behavioral performance, whilst activating multiple overlapping neural networks associated with cognition (largely attention and inhibition) and pain processing (Buffington et al., 2005; Glass et al., 2011; Martinsen et al., 2014). Given that this was the first study to explore cognitive control deficits in endometriosis in the AX-CPT, further research and replication by others is required to determine whether our findings can be generalized to the endometriosis population more broadly, or whether they are unique to our sample.

As hypothesized, target P3b and SW amplitudes were reduced in the endometriosis compared to the HC group (Ozgocmen et al., 2003; Yoldas et al., 2003; Alanoğlu et al., 2005; Hamed et al., 2012; Tomasevic-Todorovic et al., 2015; Samartin-Veiga et al., 2019). P3b has several functional interpretations, particularly in the oddball and Go/NoGo task context [e.g., context-updating (Donchin and Coles, 1988; Brydges and Barceló, 2018), stimulus-response pattern activation and updating (Steiner et al., 2013b; Verleger et al., 2016), response monitoring (Verleger et al., 2005; Fogarty et al., 2020)], yet is under-explored in cued CPT tasks such as the AX-CPT. This is the same for SW, which has been linked to response evaluation and preparation of motor responses for upcoming trials (Rohrbaugh et al., 1978; Desmedt and Debecker, 1979; Friedman, 1984; García-Larrea and Cézanne-Bert, 1998; Fogarty et al., 2019, 2020). Taken together, findings suggest that women with endometriosis and CPP have reduced activation of neuronal networks associated with stimulus-response processing, including response execution, monitoring, and evaluation. This is most likely due to neuroplastic changes in the pathways that overlap between pain and cognitive control as a result of ongoing processing of chronic pain and cortical sensitization resulting in this altered stimulus-response processing (Glass et al., 2011; Martinsen et al., 2014).

Non-target P3a amplitudes (eP3a and lP3a) in the endometriosis group were attenuated compared to HCs, a finding in line with previous studies (Ozgocmen et al., 2003; Yoldas et al., 2003; Alanoğlu et al., 2005; Hamed et al., 2012; Tomasevic-Todorovic et al., 2015; Samartin-Veiga et al., 2019). Non-target/NoGo P3a typically peaks 250–400 ms in oddball and Go/NoGo tasks and is topographically defined by a fronto-central distribution of positive activity, as shown in the HC group, peaking at 352 ms. As with the other P3 components, P3a has not been widely explored in CPTs, however, Karamacoska et al. (2015, 2019) showed distinct non-target fronto-central P3as peaking between 250 and 350 ms in a visual cued CPT (Gordon variant). Interestingly, the P3a component appeared to “split” in the endometriosis group, with dual peaks at 332 and 416 ms for eP3a and lP3a, respectively. This separation has not been reported previously, most likely due to the majority of chronic pain studies using peak amplitude quantification techniques that do not permit the separation of temporal variance as well as PCA. Chronic pain research from other modalities has reported dysfunctional “smearing” of transcranial magnetic stimulation-induced sensorimotor cortex activity reflecting increased firing of non-specific neural populations in response to chronic pain (Furman et al., 2019). As part of the maladaptive neuroplastic response to pain in endometriosis, P3a findings here may reflect similar recruitment of additional neural resources, particularly in relation to stimulus evaluation, attentional processing, and/or perhaps most of all, inhibitory demands (Bruin and Wijers, 2002; Donkers and Van Boxtel, 2004; Ramautar et al., 2004; Polich, 2007; Smith et al., 2008). Although there was no difference in task performance between groups that would reflect behavioral inhibition difficulties (e.g., commission errors), as with other studies (Glass et al., 2011; Veldhuijzen et al., 2012; Berryman et al., 2014; Masiliünas et al., 2017), P3a findings indicate that a wide range of neuronal substrates of inhibitory control are less active overall in women with endometriosis and CPP compared to HCs. No differences were noted for the non-target SW.

Reduced non-target eP3a (large effect size), lP3a and SW (each medium effect sizes) amplitudes were each associated with greater pain severity across groups, with non-target P3a showing the strongest relationship. These findings are in line with previous studies (Tomasevic-Todorovic et al., 2015), and point to non-target P3 component amplitudes as a potential objective biomarker of the subjective pain experience of women with endometriosis associated CPP. The inverse relationship between pain severity and the non-target (but not target) P3 components is most likely due to chronic pain processing taking up “bandwidth” in cognitive control networks, decreasing the availability of neuronal resources for cognitive demands associated with attention, evaluation, and inhibition of a motor response. There were no correlations between target P3b, SW and pain severity, and none of the target or non-target P3 amplitudes correlated with pain duration, the latter of which is similar to past studies (Alanoğlu et al., 2005).

### Strengths and Limitations

This study was the first to investigate the neuronal correlates of cognitive control in women with endometriosis associated CPP and provides an important contribution to the limited literature on the use of ERPs to assess cognitive control in chronic pain. This study was original in its use of the AX-CPT paradigm to assess cognitive control and the application of PCA to quantify ERP component amplitudes, which resulted in the disentangling of two temporally distinct and substantial non-target P3a components in the endometriosis group. Our study was adequately powered and the sample size was similar to other studies (Tandon and Kumar, 1993; Larbig et al., 1996; De Mirci and Savas, 2002; Ozgocmen et al., 2003; Yoldas et al., 2003; Karl et al., 2004; Alanoğlu et al., 2005; Hamed et al., 2012; Tomasevic-Todorovic et al., 2015). The findings have clinical relevance as they demonstrate that endometriosis has broader impact on pathophysiology, extending to the central nervous system (Brawn et al., 2014). In our particular sample, it seems that women with endometriosis are able to neuronally compensate for chronic-pain related changes in the brain, as demonstrated by unimpaired behavioral responses to the AX-CPT. However, the threshold for these neuroplastic adaptations are not yet known, nor is their relationship with staging of endometriosis pathology, the interaction with the peripheral nervous system, and any potential relationship with quality of life and the broader impacts of endometriosis (e.g., mental health, social and work life, finances etc.); further investigation is required to determine clinical applications (Zondervan et al., 2018).

There are a few important limitations to consider that may impact the generalisability of results. For ethical reasons, the participants with endometriosis undertook treatment as usual, which meant that pharmacological management of symptoms (e.g., analgesics) and comorbidities together with their medical management were not controlled (see Table 1), and may have contributed unwanted variability to the results; for example, one participant was taking methylphenidate for the treatment of ADHD, which is known to modulate ERPs (Klorman et al., 1979, 1983; Coons et al., 1981; Chapman et al., 1982; Lawrence et al., 2005). It should be noted that the high prevalence of co-morbidities in endometriosis is common/typical and difficult to control suggesting that our sample is representative of the population (Sinaii et al., 2002; Tietjen et al., 2007; Sepulcri and do Amaral, 2009), and the majority of other chronic pain research has not controlled for such comorbidities (Tandon and Kumar, 1993; Larbig et al., 1996; De Mirci and Savas, 2002; Ozgocmen et al., 2003; Yoldas et al., 2003; Karl et al., 2004; Alanoğlu et al., 2005; Hamed et al., 2012; Tomasevic-Todorovic et al., 2015). We also did not collect data on years of education, which may affect cognitive ability, and should be controlled for in future studies. Time of testing throughout the day was not controlled as flexibility was required to fit in with the busy schedules of the participants who were working, studying, had caring commitments etc. Further, the two-group cross-sectional design of the current study meant that it is impossible to disentangle whether the results are specifically associated with endometriosis pathology and/or CPP. However, the P3 component differences observed do not appear to be condition-dependent, as our results replicate studies investigating a broad range of chronic pain conditions (Dick et al., 2002; Weiner et al., 2006; Walteros et al., 2011; Berryman et al., 2014). Future studies should consider comparing a third group with differing chronic pain etiology and/or a sample of women with endometriosis who are not currently experiencing CPP to determine the driver of the current results. On this point, replication in a longitudinal study is essential given the limitations of cross-sectional research and the inability to draw conclusions regarding causality. In addition, acute pain (during testing) was not controlled for or monitored in this study, yet this is known to attenuate P3 amplitudes (Rosenfeld and Kim, 1991; Lorenz and Bromm, 1997; Houlihan et al., 2004). For example, past research has shown that participants with chronic pain who were not experiencing acute pain during testing (De Mirci and Savas, 2002) showed no reduction in P3 amplitudes. Future chronic pain studies could control for acute pain during testing with a simple visual analog scale to avoid this potential confound.

### Conclusion

This study was the first to explore the relationship between CPP and the neuronal correlates of cognitive control in women with endometriosis. It was found that whilst behavioral performance did not differ between the groups, target (P3b, SW) and non-target (eP3a and lP3a) P3 component amplitudes were significantly reduced in the endometriosis group relative to the HC group, and non-target eP3a, lP3a, and SW each correlated inversely with pain severity scores across groups. These findings are indicative of alterations in stimulus-response processing and inhibitory control at a neuronal level in women with endometriosis and CPP, although not necessarily at a behavioral level, most likely due to compensatory neuroplastic changes in overlapping cognitive and pain networks. Due to the detrimental impact of chronic pain on women with endometriosis, it is crucial to better understand its etiology, precipitating factors, and neurobiological implications. This study makes an important contribution to the scant literature on endometriosis and CPP, moving the field closer to a better understanding of how chronic pain affects cognitive control in women with endometriosis.

## Data Availability Statement

The raw data supporting the conclusions of this article will be made available by the authors, upon reasonable request.

## Ethics Statement

The studies involving human participants were reviewed and approved by Western Sydney University Human Research Ethics Committee and the Combined Illawarra Area Health/University of Wollongong Human Research Ethics Committee. The patients/participants provided their written informed consent to participate in this study.

## Author Contributions

GS, RB, and MA conceptualized the study, oversaw its implementation, and supervised KW, SL, and AC together with FD. GS analyzed the data, created the figures, and drafted the manuscript. RB provided input on design and analysis and provided technical assistance throughout, together with FD. KW and SL recruited the healthy control group and collected the data, and AC the endometriosis group. JF wrote data processing scripts and supervised KW to process the data and also conducted a preliminary literature review and analysis of the data. JF and AC assisted with finalizing the manuscript with GS. MA also obtained the funding to recruit the endometriosis group. All authors contributed to editing and approved the final manuscript.

## Conflict of Interest

As a medical research institute, NICM Health Research Institute receives research grants and donations from foundations, universities, government agencies, individuals, and industry. Sponsors and donors provide untied funding for work to advance the vision and mission of the Institute. The project that is the subject of this article was not undertaken as part of a contractual relationship with any organization other than the funding declared above. It should also be noted that NICM conducts clinical trials relevant to this topic area, for which further details can be provided on request. MA reports being a member of the clinical advisory committee for Endometriosis Australia and an ESIG member of Endometriosis NZ. The remaining authors declare that the research was conducted in the absence of any commercial or financial relationships that could be construed as a potential conflict of interest.
